# Experimental Biointegration of a Titanium Implant in Delayed Mandibular Reconstruction

**DOI:** 10.3390/jpm10010006

**Published:** 2020-02-03

**Authors:** Alexander Dolgolev, Igor Reshetov, Dmitry Svyatoslavov, Mikhail Sinelnikov, Konstantin Kudrin, Vladimir Dub, Vladimir Put, Vladimir Anikin

**Affiliations:** 1 Department of General and Pediatric Dentistry, Stavropol State Medical University, 310 Mira str., Stavropol 355017, Russia; dolgalev@dolgalev.pro; 2 Department of Oncology and Reconstructive Surgery, Sechenov University, Ministry of Health of Russia, Bolshaya Pirogovskaya, 6/1, Moscow 119431, Russia; reshetoviv@mail.ru (I.R.); prof@putimplant.ru (V.P.); 3 Institute for Regenerative Medicine, Sechenov University, Ministry of Health of Russia, Bolshaya Pirogovskaya, 6/1, Moscow 119431, Russia; dssvyatoslavov78@mail.ru; 4 Department of Oncology and Plastic Surgery, Institute for Advanced Studies of the Federal Medical-Biological Agency, Volokolamskoye Shosse, 30, Moscow 123182, Russia; kudrin_k@rambler.ru (K.K.); anikinvladimir@hotmail.com (V.A.); 5 Joint Stock Engineering Company Atomstroyexport JSC “Science and Innovations”, Kadashevskaya Naberegnaya, 32/2, Moscow 115035, Russia; vladimir.dvchem@gmail.com

**Keywords:** mandibular reconstruction, personalized implants, 3D implant modeling, medical technologies

## Abstract

**Background**: Mandibular reconstruction, after extensive resection of the mandible for the treatment of oral cancer, is a well-known procedure, however, relatively little is known about bone integration into the titanium implant after reconstruction with a temporary plastic implant. The main goal of this experimental study was to study the process of osseous integration into the titanium implant in an in vivo experiment following prior mandibular reconstruction with a temporary plastic implant. **Materials and Methods**: Four ewes initially underwent a partial one-sided resection of the mandible, with the formation of an approximately 3 × 1 cm defect. All of the subjects received reconstruction with an implantation of a plastic plate (3 cm). The plastic plate was removed and replaced by a titanium implant at 1, 3, 6, and 12 months, accordingly. Both plastic and titanium implants were made via 3D-printing technology and personalized modeling. A total of 6 months after titanium implantation, a histological evaluation of biointegration was performed. **Results**: All surgeries were uncomplicated. The integration of osseous tissue into the titanium implant was seen in all cases. Histologically, each case showed variable integration of dense fibrotic tissue with fibroblasts and non-mature bone tissue with a definitive layer of bone matrix with many osteoblasts on the periphery. The prior implantation of the plastic plate did not interfere with bone integration into the titanium implant. **Conclusion**: Preliminary results demonstrated that a temporary plastic implant for mandibular reconstruction does not interfere with the consequent osseous biointegration of a permanent titanium implant. This shows that temporary reconstruction is a safe solution when delayed mandibular reconstruction is required due to disease severity.

## 1. Introduction

Mandibular defects after extensive resection, due to tumors and other pathology, are quite disfiguring and can lead to significant cosmetic and functional deficiency. The reconstruction of the resected mandible could be performed with free bone grafts or a variety of implantable constructions. Porous titanium is quickly becoming the material of choice to manufacture complex implants for the replacement of resected mandible tissue, as it is very similar in structure and mechanical features to bones. Another advantage of porous titanium is the possibility of creating complex anatomically correct constructions using modern 3D printing technology and applying them clinically.

Reconstructive surgery of the mandible and other osseous structures of the skull requires well developed techniques for the reparation of postoperative defects after extensive resection. These methods include mandibular reconstruction using free or vascularized bone grafts alone or in combination with vascularized soft tissue flaps with or without integration of a metal implant. All these methods carry the risk of delayed infection, the resorption of cadaveric bone grafts, and difficulties in patient rehabilitation. 

Current progress in digital diagnostics and the computerized planning of treatment provides a wide variety of options presented by 3D printing using different materials. These technologies provide a new opportunity in the surgical planning and reconstructive intervention of complex patients with individual implants.

There are a few case reports, clinical studies, and experimental studies regarding the use of porous titanium implants in the reconstruction of the mandible as primary surgery [1]. The main problem with implantable structures for the reconstruction of mandibular defects is the need for secondary revisional procedures, which undermine the efficacy of one-stage reconstructive surgery [2,3]. The efficacy of mandibular reconstruction with direct implant prosthesis is a controversial topic [4], yet, in cancer patients with aggravating comorbidities, the best solution can be achieved by individually printed prosthetic mandibular implants [5]. The osseointegrative capabilities of modern porous titanium implants is high, and current technological manufacturing progress, including selective laser melting and 3D printing, has increased the interest of surgical teams in implant-based reconstruction. 

The choice of material for implant-based reconstruction is dependent on the similarities between implant and bone physio-functional characteristics. Porous implants have been appraised for their high biocompatibility and biointegration potential [6]. While maintaining high integration into the surrounding osseous tissue, the porous implant provides stable strength and load-bearing characteristics, similar to that of the bone itself [7]. Despite the proven significance of exogenous materials in mandibular reconstruction, their use is often limited in cases where secondary surgical intervention may be required.

Based on our experience and literature data, annealing and selective laser melting (SLM) provide the best physicochemical characteristics when preparing an individualized titanium implant. The implant was prepared under annealing temperatures ranging between 625–725 °C. Annealing in this interval led to an increase in plasticity after SLM of up to 16%. Annealing at 1 hour with exposition at 675 °C allows the titanium implant sample to achieve the required physicochemical characteristics and correspond to wrought titanium (GOST R ISO 5832-2, Grade 4). The porous structures that only underwent SLM showed a tendency to deviate from the original cubical form due to the weakness of the unannealed titanium structural component and the occurrence of local thermal stresses [8].

Occasionally, in clinical surgical oncology, the resection margin may not be reliably assessed at the time of surgery, mandating the necessity to perform secondary resections [9]. In these cases, positioning a permanent implant would be hazardous, because it would jeopardize secondary surgery if it becomes indicated. In a situation like this, one of the options would be to implant a temporary prosthesis and, if the absence of tumor in the surgical margin is confirmed, it would be replaced by a permanent titanium prosthesis. If tumor growth is detected in the surgical margin in the final histological examination, an additional resection may be undertaken, this time using a permanent installation of a titanium implant. 

The biointegration of a secondary implant is possible, according to several clinical and experimental studies. In 2001, Persson et al. created an artificial peri-implantitis around a titanium implant in the mandible, and, even in these conditions, it was possible to establish a substantial reintegration of osseous tissue in a rough surface implant (unlike the smooth surface implant) [10]. The significance of implant surface texture for bone apposition was confirmed in the study of Lai et al. (2009) [11]. Namgoong et al. (2015) investigated bone formation and integration following the surgical treatment of experimental peri-implantitis on experimental beagle dogs. Their study demonstrated significant osseous growth and biointegration in blasted/acid-edged implants with a hydroxyapatite nano-coated surface [12]. In clinical settings, authors Wei et al. (2003) used the titanium implant reconstruction of mandibular defects in combination with different methods of soft tissue reconstruction. Late exposure was observed in 46.15% of cases, one third of patients required secondary salvage reconstruction. This shows that titanium mandibular implants, particularly in complex surgeries, remain controversial. This remains an important topic of discussion [13].

More recently, Qassemyar et al. (2017) demonstrated successful healing after custom made porous titanium implants were used in patients not amenable for bone free flaps [14]. In an experimental study by Wetzel et al. (1999), the authors induced peri-implantitis by placing implant-to-bone silk ligatures. The potential healing of peri-implant infections with bone formation was proven after the infection was controlled, however, true reintegration of osseous tissue appears to be more difficult to achieve [15]. In a series of complex cases of mandibular reconstruction, Mariani et al. (2006) found no difference in the success rate between titanium and stainless-steel plates. However, most of the publications are related to dental implants and not to large metal constructions used to replace the resected part of the mandible [16].

No significant scientific input into investigating bone integration in the titanium implant positioned after previous surgical trauma or after explanting a temporary prosthesis has been carried out. Many questions remain unsolved, such as the implant adaptation to the defect edges, the reliability of prosthesis integration with osseous tissue after secondary implantation. The main goal of this experimental study was to assess osseous integration into a porous titanium implant in an animal model after the resection of the mandible and the implantation and successive removal of a temporary prosthesis.

## 2. Materials and Methods

**Figure 1:**
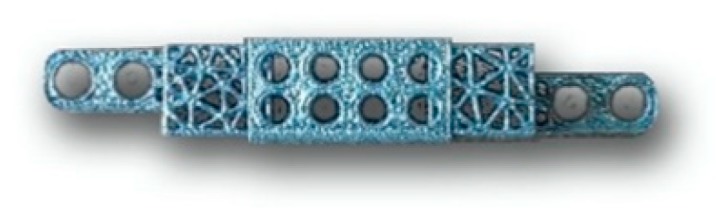
Model of a bionic titanium implant.

Material characteristics. Implants of titanium bionic structures were developed with an attempt to imitate the normal structure of the bone (Figure 1). The pore size of these titanium implants was about 600 microns and the trabecular diameter was 300 microns. The pores were tetragonal in shape. The main reason for the development of this particular titanium implant structure was an attempt to promote osseous biointegration of the porous material with trabecular bones, maintaining high porosity (exceeding 70%) and a low coefficient of resistance (1.5 GPa). Samples were printed on a Russian-made 3D-printer for layered printing via the selective laser melting (SLM) of metallic powdered materials (MeltMaster3D developed by Rosatom). Titanium VT1-00 metallic powder was used for production. We used an annealing protocol for implant preparation with exposition at 675 °C for 1 h.

Experimental model. Four adult ewes of North Caucasian breed aged between 1.5 to 2 years were used for the experiment. All animals were kept in an animal shelter at the Russian Institution of Sheep and Goat Breeding. All surgical interventions were performed in the Department of Experimental Medicine of Stavropol State University by the same surgical brigade (two surgeons). This research project was approved by the local ethics committee and all experiments were performed according to the rules of laboratory practice of the Russian Federation and standards of Good Laboratory Practice. 

**Figure 2:**
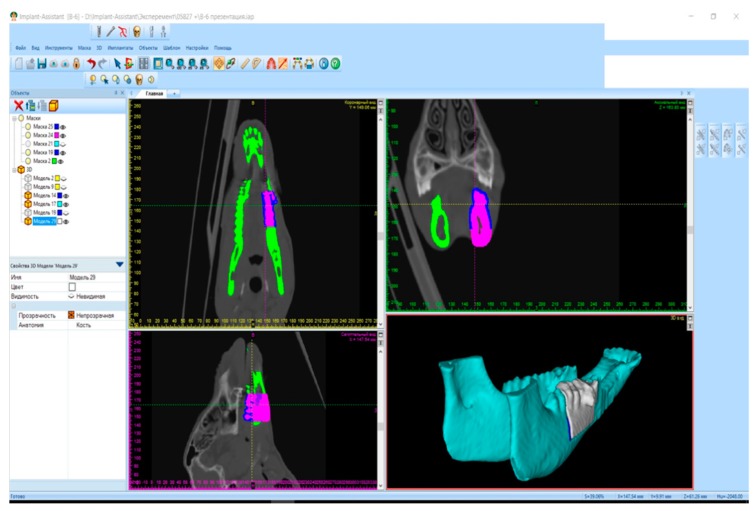
Modeling of titanium implant via “Implant Assistant”.

Implant preparation. Surgical planning included the multi-spiral computerized tomography charting of the mandible and the creation of individual mandibular gypsum mould models. These models were scanned using an external scanner and computerized via “Implant Assistant” software in order to create a digital prototype of the implant. This program performed a three-dimensional reconstruction of the mandible and teeth from the computerized tomography data. As a result, a three-dimensional digital prototype was created in each case. Modeling of the created digital prototype was performed. A 3D printer was used to create the plastic and titanium implants based on the digital prototype (Figure 2). For the printing of titanium implants, SLM 3D-printing technology was used.

Surgical protocol. All manipulations on animals were performed under general anesthesia using an intramuscular injection of Sodium Thiopentone (50 mg per kilogram) with Droperidol premedication (0.2 mL per kilogram) and Midozolam 0.5% (0.2 mL per kilogram). All implants were sterile. Sterilization was performed in Getinge HS6617 ER2 autoclaves (Sweden), Pl sterilization program (indicator 6, exposition at 134 °C for 5 min).

**Figure 3:**
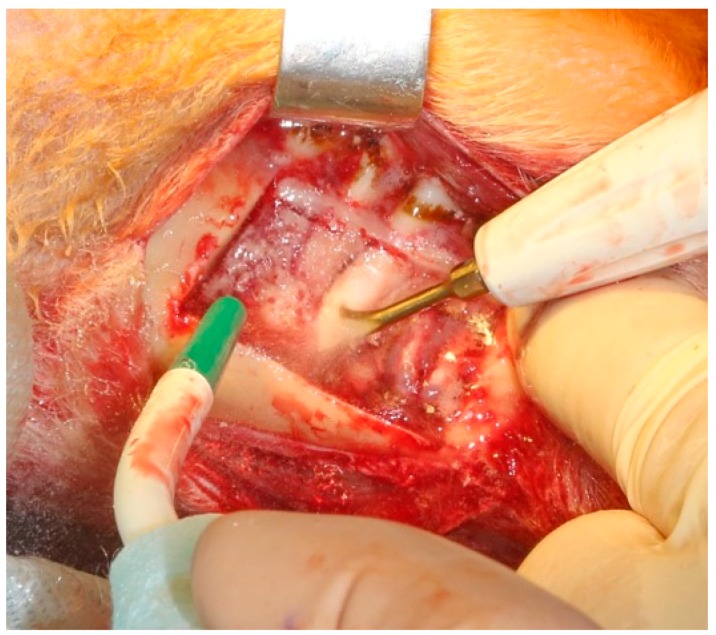
Surgical preparation of implant recipient zone in the mandible.

Surgical intervention began with a right-sided 6–7 cm incision on the lower jaw of the animal subject in order to expose the body of the mandible. The experiment protocol included the primary resection of the external cortical layer of the mandible at an extent of 3 cm (Figure 3), with consequent implantation of a sterilized plastic implant created via 3D printing. 

A plastic implant was positioned into the mandibular defect and secured with standard orthopedic screws. Layered wound closure was performed with absorbable sutures. The skin was closed with non-absorbable material. This completed the first surgical stage of the experiment. As a result, all subjects received 3D-modeled plastic implant for mandibular reconstruction.

**Figure 4:**
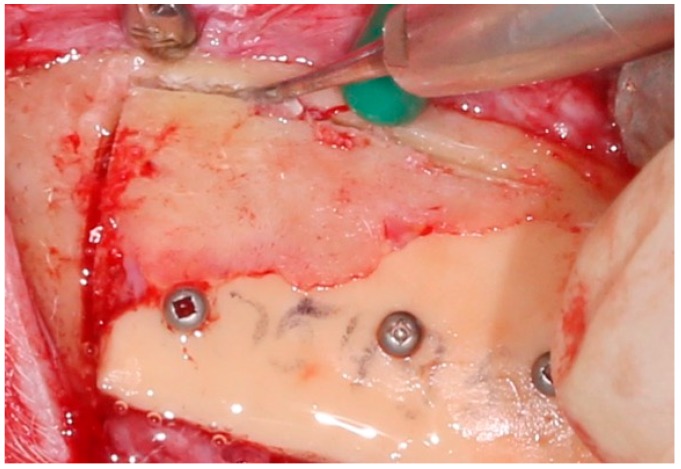
Excision of the plastic implant three months after primary surgical intervention.

Secondary surgical intervention occurred for each subject in a different timeframe. Th extraction of the plastic implants was performed at 1, 3, 6, and 12 months after installation and was replaced with an individualized titanium implant into the bed of the removed plastic implant. The original surgical scar was excised and the previously positioned plastic implant was exposed and excised with a piezoelectric scalpel. Tissue samples were taken for histological evaluation (Figure 4).

**Figure 5:**
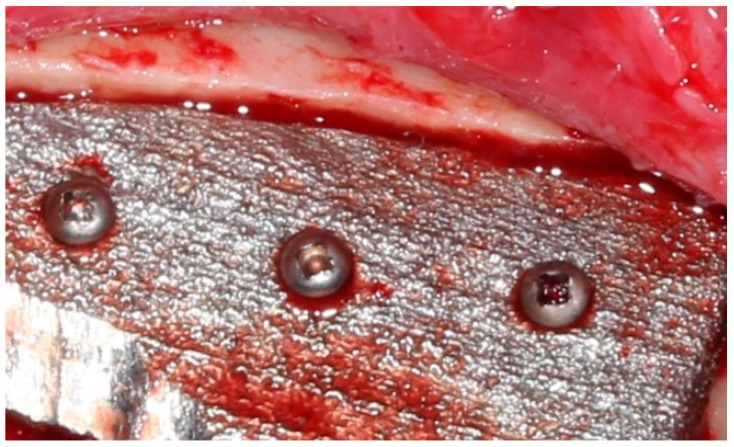
Secondary mandible reconstruction with porous titanium implant.

The previously printed porous titanium implant was then positioned and secured with standard screws into the area of the explanted plastic implant (Figure 5). Wound closure was then performed. 

All experimental subjects were returned to their usual environment within 24 h and were allowed to eat normally. The stitches were removed 10 days after surgical intervention. All animals were withdrawn from the experiment 6 months after secondary surgery and underwent euthanasia due to the risk of continuing adverse effects. 

Histological evaluation. A total of 6 months after secondary surgery, after euthanasia, the reconstructed mandible underwent histological evaluation. The bone was excised using an osteotome and bone nibblers around the implant. Bone specimens were fixed in 10% of Neutral Formalin for 10 days. Subsequently, all fragments were decalcified with 3-Chlorocetic acid. After decalcification, all samples were washed in 96% Ethanol for 3–4 days and embedded in Paraffin. Standard microtomes were used to perform histological slices 5–5 microns stained with Hematoxylin and Eosin, Picric, and Fuchsin acid (Van Gieson’s stain). Toluidine Blue and Periodic acid-stiff staining were also performed. The analysis and structuration of the results was carried out following the final experimental data acquirement. Microscopic photographs were acquired through a camera mounted on the microscope.

## 3. Results

All experimental animals demonstrated no evidence of discomfort after the primary or secondary surgery. There was no evidence of local inflammation in the area of surgical intervention or the site of the installation of plastic and titanium implants. Following the implantation of the plastic plate, within one month, there was no local inflammatory response with some excess of soft tissue near the implant edge. At three months there was a visible excess of bone tissue covering the surface of the plastic implant. Extraction at 6 months showed significant osseous tissue covering the implant surface.

**Figure 6:**
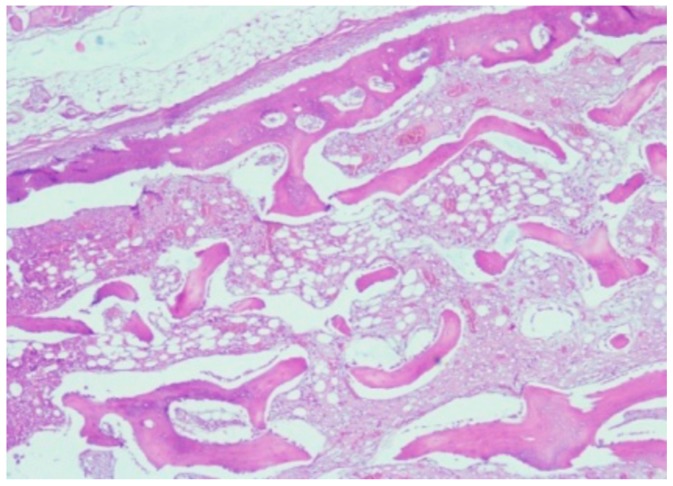
Osseous integration of titanium plate implanted into a partially resected mandible.

Following 12 months after secondary surgery, the extracted titanium implant showed notable osseous tissue integration into the pores of the titanium implants as well as above and over the implant edges. The edges of the implant were therefore firmly embedded into surrounding osseous and scar tissue (Figure 6).

**Figure 7:**
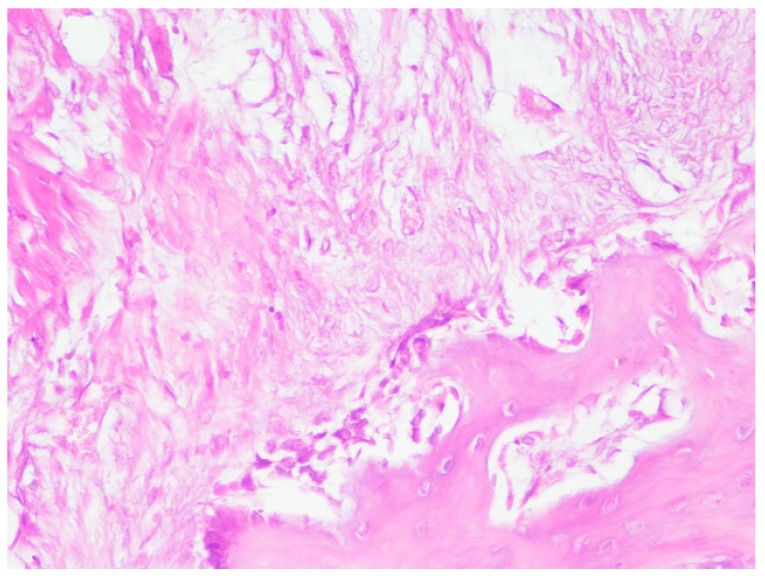
Reactive osteogenesis near the titanium implant.

Histological examination of osseous tissue around the orthopedic screws demonstrated no evidence of granulomatous inflammation. In the area close to the implant there was a layer of dense fibrotic tissue with fibroblasts and fibrotic tissue with a trabecular structure and non-mature osseous tissue with loop-like structures (Figure 7).

**Figure 8:**
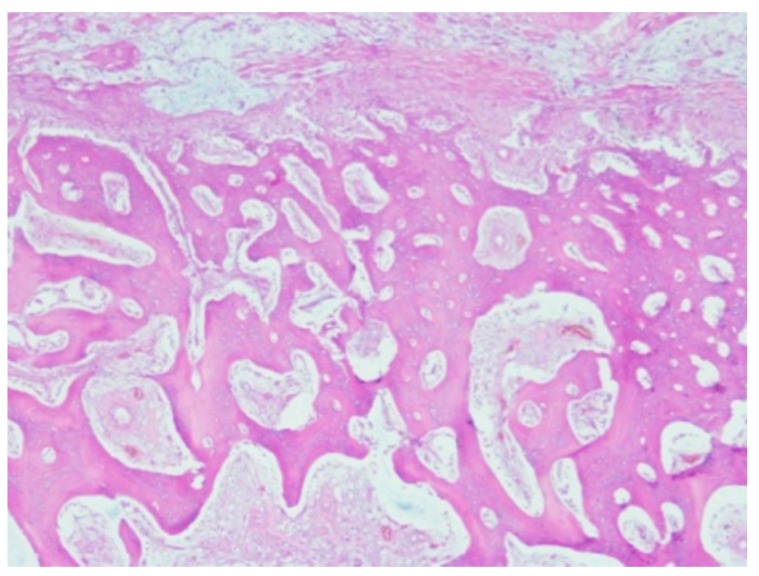
Fibrosis and maturing bone near the titanium implant.

A definitive layer of osseous tissue, with many visible osteoblasts on the periphery, surrounded the implant edge. The osteoblasts had a typical cylindric form with some large dark nuclei. No endochondral ossification was noted. Multiple osteoblasts were seen close to the new bone trabeculae. There was reticular tissue growing between the trabecular space with evidence of neoangiogenesis (Figure 8).

## 4. Discussion

Extensive surgical excision with subsequent reconstruction remains the mainstream technique to treat complex tumors of the head and neck. Bone reconstruction could be performed with free bone grafts, by using various synthetic implant materials as well as carbon-based materials, or by using a combination of these methods. The use of porous titanium implants is favorable because this material, based on structural, physical, and mechanical characteristics, is very close to osseous tissue, which allows for quick and reliable biointegration between the remnant of the bone and the porous implant.

The use of titanium in medicine, especially for the personalized creation of implants, is now highly appraised. Titanium is extremely hypoallergenic and has a very high biocompatibility. It is possible to use titanium in almost every situation in which implants are required to replace the missing part of the bone. What is particularly important is the possibility to use 3D printing technology to create biocompatible implants with features close to the natural bone. Modern technological advancement allows for the creation of complex titanium constructions with the use of digital computer planning and 3D-printing to manufacture individualized implants. The poor adaptation of implants to the area of the defect, the stability of the implant, and the erosion of the bone remain problematic. Even though the number of studies regarding osseous integration between the titanium implant and the bone is increasing, very little is known about using titanium implants for secondary reconstructive surgery. 

It is a well-known fact that after partial facial skeleton resection it is not always possible to determine whether the tumor was completely resected. In this case, it is necessary to occasionally implant a temporary plastic implant with a plan to subsequently remove it and insert a permanent titanium prosthesis. In our experimental study, it was clearly established that the positioning of a temporary plastic implant into a mandibular defect led to the growth of osseous tissue over the plastic implant. The process of osseous integration and growth continued with titanium implants after the plastic implants were replaced. What is particularly important is that there was no inflammation detected on the interface between the implant and the recipient zone in the resected bone. 

In all cases, the direct contact of osseous tissue with the titanium implant was established, which confirms the biointegration of the metal implant into the mandibular tissue. There were also areas of reorganization of osseous and soft tissue on the surface between the implant and bone with evidence of fibrous and bone integration into the implant. 

The authors conclude that the data obtained demonstrates that a porous titanium implant used in secondary surgical reconstruction provides significant osseous biointegration. This shows that it is possible and often advisable to postpone the implantation of a permanent implant in order to achieve better therapeutic results and account for possible complications after tumor resection.
